# Supporting global health goals with information and communications technology

**DOI:** 10.1080/16549716.2017.1321904

**Published:** 2017-08-25

**Authors:** Magnus Boman, Erik Kruse

**Affiliations:** ^a^ KTH/ICT/SCS, Kista, Sweden; ^b^ RISE SICS AB, Kista, Sweden; ^c^ Ericsson, Kista, Sweden

**Keywords:** Health data, health data analytics, precision medicine, connectibility, privilege, sensemaking

## Abstract

The objective of this study is to critically assess the possible roles of information and communications technology (ICT) in supporting global health goals. This is done by considering privilege and connectibility. In short, ICT can contribute by providing health information via four different kinds of access, each with its own history and prospective future. All four are analyzed here, in two perspectives: business-as-usual and disruptive. Health data analytics is difficult since the digital representation of past, current, and future health information is lacking. The flow of analytics that may prove beneficial to the individual and not just meet abstract population-level goals or ambitions is analyzed in detail. Sensemaking is also needed, to meet the minimum requirement of making prospective future services understandable to policymakers. Drivers as well as barriers for areas in which policy decisions have the potential to drive positive developments for meeting the Sustainable Development Goals are identified.

## Background

Supporting global health goals with information and communications technology (ICT) involves four kinds of access, namely access to the Internet, to individual health data (medical data), to individual data indirectly linked to health, and to data about the environment of the individual, relevant to health. These are, in turn, chiefly accessed today via a computer or smartphone, a caregiver, a personal health or lifestyle adviser, and no one.

Internet access provides information over the full spectrum of trust, from scientifically proven results to rumors. It is also fundamental, as it is an element of the other three types of access. Trust spectra exist in all four types, and are not only propagated from Internet access: a caregiver may be informal, such as a parent or a first responder at the site of an accident, or formal, such as a surgeon. An adviser may be an a personal trainer, a yoga instructor, a self-appointed expert on a new diet scheme, or even a guru. Even if, in most cases, no one can provide sound advice on health-relevant data, the media tries hard to do this. And a house broker (estate agent) may argue that particulate dust from a nearby highway does not pose a threat to quality of life, since trustworthy air-flow analyses show that the particles are light enough to travel far beyond the house, which is, however, fitted with state-of-the-art air filters as a precaution.

### Health data analytics is difficult

Policymakers and decision makers are often presented with massive data sets that they cannot analyze themselves, but which are said to have value potential. In some cases, they are instead presented with complicated finished analyses of massive data sets, and so again fail to create value from the input. This problem is becoming increasingly common and global, and it leads in some cases to a feeling of missed opportunities and to stress caused by not being able to follow the technological and methodological development of analytics. This is due to a lack of competence, and such competence is currently difficult to secure [1]. In the health domain, complex interactions between multiple determinants of health and well-being (age, gender, lifestyle, behavior, social and physical environment, etc.) are not well understood. In particular, co-morbidities are a challenge [2]. New ICT allows for health data aggregated at the population level to be complemented by data from individuals, via wide-scope health data collection that is potentially accessible in all four ways defined above. Best practice among clinicians must be respected at all times if ICT is going to contribute to the most important of goals, namely to improve health globally.

Privileged people today can look forward to precision medicine, with the former president of the USA handing out dedicated funds for research and development [3], and the European Commission doing the same [4]. The value here lies in going from macro (where the same diagnosis results in the same substances being prescribed) to micro (where the substance depends on the individual’s medical history, and even his or her physical and social environment). Many of the data used for precision medicine are not health data *per se*. Instead, they pertain to lifestyle and the cultural, geographic, economic, and political environment. The Internet of Things (IoT) and related technological developments on the horizon rest on the proviso that sensors can amass data on each individual’s lifestyle and environment dynamically and at low cost. The exact value proposition, though, is still unclear. In the example of the house broker above, the IoT may well be flagged as a new technology for keeping the house owner safe by dynamically sensing particles in the indoor air. In truth, the particles most dangerous to humans are small enough to pass through any cheap IoT sensor undetected.

Underprivileged people given Internet access can receive important information about a disease or symptom, or connect with others suffering from similar problems, directly supporting targets as part of the Sustainable Development Goals (SDGs), i.e. SDG3: Good health and wellbeing [5]. However, the other three kinds of access will probably take time, much more than a decade, to realize. Existing inequalities in the social, economic, political, and technological perspectives will persist and be emphasized in issues concerning health [6]. That said, advances in ICT for the privileged have historically proved globally significant, sometimes with a quicker uptake among the underprivileged, as in the case of telemedicine. Globally, the most widely used means of accessing the Internet is through mobile networks and a mobile device. Tracking, storing, recalling, and making good use of mobile data is therefore key, albeit difficult in view of constantly changing regulations and directives.

### Very few health data are digitized

Access to the Internet, in some cases, also means access to the other three kinds of data mentioned above. But even among the most privileged, with continuous and ubiquitous net access (‘always on’), some individual health data are offline. Notably, unless a person has serendipitously ended up in a longitudinal research study, very few historical health data are available. Old X-rays and scans are rarely digitized and most analog records are likely to have been destroyed. What happened when a person was born may still reside in the memory of their parents, but as this memory fades, facts become lost over time. A person’s blood pressure during and just after a 10 km race run an hour ago will likewise not be on the Internet, unless a device with cloud-logging functionality was brought along. The number of emails received and the number of missed calls while out running are examples of individual data only indirectly linked to the health of an individual. These are obtainable from the network, but the neighbor ringing the doorbell while the person was away typically is not. Environmental data points include the temperature during the run, possibly logged or derivable after the fact, while the quality of indoor air upon returning home is likely to remain unknown.

For people without even sporadic Internet access, including those residing in the parts of the world with the lowest percentage of coverage and smartphone subscriptions, even more health data are analog. These individuals could be considered off-grid, although general practitioners (GPs), primary care workers, and first responders in extreme situations may transfer their electronic health records or other health information to digital and sometimes searchable data. In these regions, and to this global population stratum, reliance on regional or national open data is much more important. Even if open data cannot be accessed at the individual level, for reasons of privacy or regulations, population data, statistics, and general health information can be of vital importance to an individual trying to make the right choices to stay safe. Macro-level information on the weather, air and water pollution, disease outbreaks, and so on can be equally important. Data science now uses methods that can merge and synchronize disparate data sets without resorting to data warehousing or similar established data-analytics methods. Such data sets include patient self-reporting questionnaires submitted over various modalities, socio-demographic data, and molecular data [7], and may be sourced from distributed sources employing many groups of people and reporting modalities [8].

### Objectives

After explaining the methods employed, what follows below is an analysis of the prospects for ICT to be of instrumental use to the pursuit of global health goals, i.e. the main objective. The analysis rests on the four kinds of access to health information listed in the Background section above. Two main scenarios, i.e. business-as-usual and disruptive, are first separated, and then compared in the Discussion section, before conclusions are presented. The analysis reflects a second objective: to illustrate the difficulties in using ICT to support global health goals. This is addressed by considering barriers to ICT adoption and access, and also by illustrating by example the difference between micro and macro, including the difference between individual value and population-level goals.

## Methods

The large uncertainty regarding the future of mobile-based Internet access, and the value of the mobile health (mHealth) services accessed motivate a prospective analysis. The present analysis rests on Ericsson Mobility Report data for the business-as-usual case, on primary data gathered for a currently running ICT foresight project for the disruptive scenario, and on knowledge resulting from direct participation in several current research projects on using data science to solve clinical health problems. Since the area is well established, a deductive literature study was completed. The academic full-text archive available from KTH was searched for keywords, e.g. ‘sensemaking’, ‘micro’ AND ‘macro’, and ‘precision medicine’. Titles and abstracts were then browsed and relevant papers read. In addition, relevant knowledge repositories built by the European Commission ‘EIT Digital Innovation Radar’ and those of two other proprietary radar/outlook forums were scanned for trends, outlooks, horizon scans, and projections. A few semi-structured interviews were conducted with industry professionals, and preliminary results were discussed with a panel of two experts on foresight and one retired medical science writer. The research approach is quantitative, but since the disruptive aspects deal with perceived user values there are also qualitative aspects, such as feeling either relaxed or stressed out from being always on. The qualitative aspects were dealt with in the semi-structured interviews and were also discussed with an independent clinician, representing the caregiver stakeholder. Anecdotes were collected from a field study in southern India, conducted in February 2017.

## Results

### Business-as-usual scenario

In a business-as-usual scenario, Ericsson forecasts that by 2021, the world’s mobile networks will constitute a technology base with the capability to provide Internet access to 7.2 billion people, or 92% of the global population. The rapid uptake and evolution of mobile technology, as well as the declining cost of smartphones, particularly in developing countries, are expected to be the two key drivers of this dramatic increase in access to the Internet. Today, almost half of the world’s population lacks access to the Internet, the Groupe Speciale Mobile Association (GSMA) stating that 3.24 billion (44%) had mobile access at the end of 2015 [9,p.8], with people in developing countries representing an overwhelming majority of the unconnected. Access to the Internet is a fundamental enabler for improving quality of life and global health goals. Infrastructure providers and telecommunications companies also see it as a critical success factor in realizing the SDGs [10], and GSMA has recently launched the app *SDGs in Action* with Project Everyone.

Today, 4.9 billion people subscribe to mobile services, with slightly more than 50% connected over a 3G or 4G network using either a smartphone or a device with mobile Internet features. The remaining mobile subscribers, approximately 1.18 billion people, are currently connected to 2G networks or use a 2G device. Although 2G provides significant value to everyday life, including some important data services, it is unreasonable to consider a 2G service as providing access to Internet in the same way that 3G, 4G, and future mobile technology generations are considered to provide Internet access. By 2021, Ericsson [11] forecasts that more than 6 billion people will be subscribers to mobile services, with the share of 3G, 4G, and 5G users sharply increasing to 86%, with only 14% of the world’s mobile users connected via 2G. In other words, by 2021, 2.7 billion more people will be connected to the Internet over mobile networks compared to today.

Ericsson’s mobile-based Internet access forecasts show slight differences from estimates from other sources such as the International Telecommunication Union (ITU) or the World Bank. Typically, the ITU, GSMA, and World Bank report a somewhat higher number of Internet users, qualifying the uncertainty inherent in these projections. An Internet connection forecast based on Ericsson Mobility Report data yields the following predictions within the 2015–2021 time horizon:

1. The number of people connected to the Internet through a 3G, 4G, or 5G device will increase from 2.6 billion to 5.3 billion.
2. The number of people connected to the mobile network with a 2G device (i.e. basic communication services but no Internet access) will decrease with continued technological momentum from today’s 2.4 billion to 0.8 billion.
3. The number of people without any mobile device will decrease from 2.3 billion to 1.7 billion.

Of the 2.3 billion people who did not have a 2G device and lacked connection to the Internet in 2015, 300 million lived in areas with 3G coverage. From a technological standpoint, this population has the possibility to connect to the Internet. By 2021, the population that lacks access to a 2G device and an Internet connection is predicted to drop to 1.7 billion, while the number of people with the potential to be connected will rise to 1.1 billion. That leaves some 600 million people, of whom 300 million lack access to the Internet and the other half live in areas with only 2G coverage. Although by late 2015, some 600 million people worldwide remained outside coverage, that figure is expected to drop to 300 million by 2021. This projection is new and based on Ericsson data [11]. Of the unconnected population, it is projected that by 2021:

1. Two out of three will live in rural areas.
2. Two out of three will be above the age of 25.
3. One out of two will not be able to afford a 3G connection.
4. One out of three will be illiterate.

A significant proportion comprises displaced populations [10]. Many are unaware of, or hesitant to use, the Internet. In addition to an absence of network access, other key factors for the unconnected population include a lack of understanding of the Internet as a concept and how it can help to improve quality of life (including personal safety and health), as well as poor (digital) literacy skills. Gaps in enabling policy frameworks also play a part [12].

Tina has a tiny clothes shop in a small town in southern India. Her customers are mostly Indian tourists that have come for honeymoon trips to the oceanside, but there are occasional foreign tourists too. Asked about Internet presence, Tina explains that they tried a Web shop but that they are now only on Facebook. Asked why she cannot spend time on the Web when the shop is quiet, Tina is first looking at her children playing just outside. Then she admits: ‘I do not read and write, and that’s important when you are on the Web’. So her Internet access is not enough, she needs a broker, and keeping a Web shop via a broker gets too complicated. Tina is in the state of Kerala, with an official literacy rate of 100%, in itself an illustration of the difference between micro and macro. She needs a broker, like a trusted family member, for her health information too. Taking norms and culture into account, this is even more difficult for Tina than trying to take her business online.

### Disruptive scenario

In a disruptive scenario, there are also two key drivers: a user-driven and an industry-driven expansion, which occur in parallel. Disruption among the privileged comes in the form of immersive and pervasive uses of the Internet, prompted by a currently foreseen but yet to be realized power of visualization and layering of the experience of reality. Not only data about the body of an individual, but his or her *sphere*, or relevant data environment, will be utilized in the future. Machine intelligence, augmented reality, assisted industry solutions, and mind interfaces are all on the list of technological service enablers [13]. Such technological advances allow for all kinds of health information to be used, while among the underprivileged, new values to the users are likely to be realized with less technology, e.g. a laptop up a flagpole, to provide Internet access via a MESH network [14]. When *The Economist* put a cell phone on the cover of the 10 March 2005 edition, with the legend *The real digital divide*, the journal stated that ‘an extra ten phones per 100 people in a typical developing country increases GDP [gross domestic product] growth by 0.6 percentage points’ [15]. A report validating and refining this later on was published by Deloitte and GSMA in 2012, based on data from 2008–2011. A 10% increase in mobile penetration leads to an increase of GDP per capita growth of 0.65%, while a 10% increase in 3G penetration increases annual GDP per capita growth by 0.15% [16]. With Internet access via low-cost smartphone enabling health services of great value to any individual or their family, such user value may be contingent on 3G or 4G access, prompting the need for a redefinition of the digital divide.

Most visions of the future, including those of the European Commission in a 2030 report, view the development of new technology as an enabler for closing the divide: ‘The resulting economies of scale will be significant, taking account of the convergence of holographic virtual reality and 5G, which will revolutionise tele-presence … All aspects of society – such as politics, governance, education, science, lifestyles, collective intelligence networks, the setting-up of open systems, and health, including transformation of the human genome – will be transformed by technological breakthroughs’ [17,p.35–36].

Innovations in the visualization of complex graphs and flows on smartphones may drive mHealth for the dissemination of health information, e.g. via a personalized app. In the case of Tina (see above), an oral natural language interface for English and Malayalam would provide immense value improvements to her daily life. While macro-level outbreak visualizations are already on the Web, dynamically updated, and zoomable and viewable on a smartphone (e.g. [18]), micro-level prevention, information, and intervention are all still underdeveloped from an ICT perspective. This may change rapidly with advances in data science, enabling the individual to stay informed on new viruses, water and air pollution, condom use, seasonal outbreaks of influenza-like illnesses and corresponding vaccine information, and much more. Hundreds of freely available apps for personalized health advice have been developed in the last year alone, in the best case making evidence-based medical research easily available to citizens.

**Figure 1.**
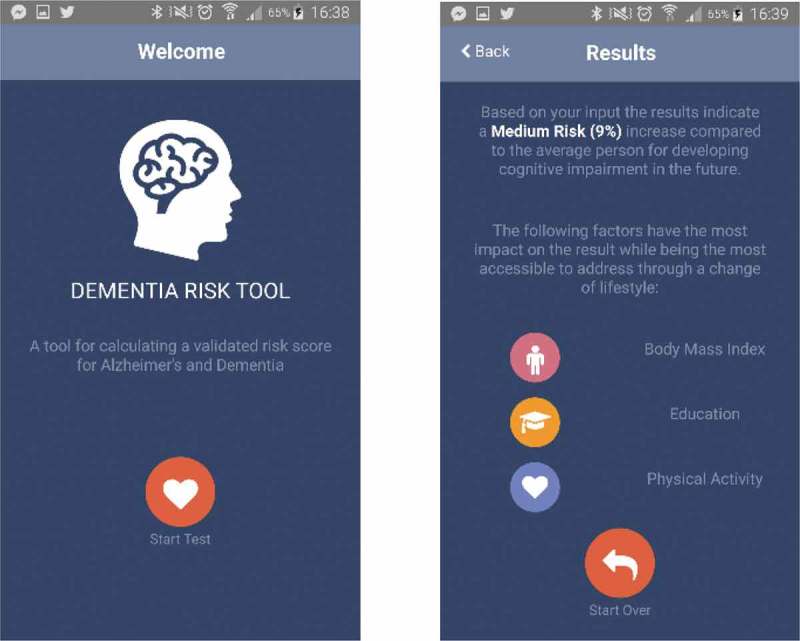
An example of a health app designed to use medical data from self-reporting (front-end analytics), as well as non-medical data from its use (back-end analytics). Screen dumps from current development version.

An example of such an app currently in development is *Dementia Risk Score*, which is built on validated research [19] on cognitive decline, memory problems, and aging. By filling out a simple questionnaire on a smartphone (Figure 1), the user can be given an individual risk score and stay informed on how to contact with caregivers, as necessary. Informed consent is given upon installing the app, allowing researchers to collect user data on the server side. By synching such data with the input user data, health information can be customized. Caregivers, such as the GP facing the user as a patient, can access data from the server side, guiding intervention. Given the huge societal costs of dementia, early detection, prevention, mitigation measures, and information on how to access help early are of value to society and individuals alike [20]. Machine learning methods can look for correlations in data, and feed some of the information obtained back to the individual and other pieces of evidence to the caregivers.

**Figure 2.**
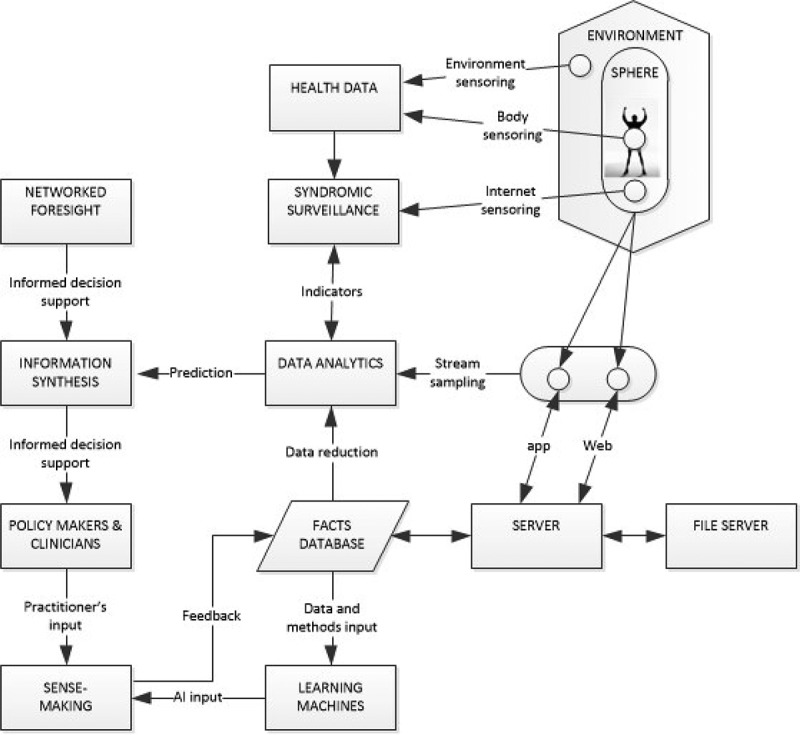
Data flows relevant to health information that feed into analytics.

To many people, a quantitative score, or a ‘traffic lights’ red–amber–green indicator, is preferable to more advanced means of measuring risk, especially in the app context. Even for a simple application, substantial steps can be taken towards understanding early signals or calming the fears of relatives, for instance. Figure 2 shows what a set-up could look like today, at a high level of abstraction. Starting from the individual, and letting the dots represent points of data collection, Internet data and health data can be stored. The sphere around the individual represents how the individual is understood from the outside, via his or her digital traces, sometimes referred to as a *data double*. Data that are entered into an app with a purpose, such as *Dementia Risk Score*, pertain mostly to lifestyle. Such data may be stream-sampled for machine learning purposes, and the app- or Web front-end is supplemented by a server back-end, again useful for artificial intelligence (AI) purposes. Finally, parts of the elusive environment data may be captured and sent on for further automated analyses.

Several provisos underlie this depiction. The individual health data are not sitting in one repository. In many countries, private and governmental caregivers keep separate and protected health records, as do other stakeholders, not least insurance companies. Some of the digital traces left from individual Internet use are impossible to track, or at least to recall. The data on the environment of the individual [4] will be partial and noisy, and probably capture less than 1% of daily life, even in the most ideal (from the perspective of health researchers) of circumstances. Syndromic surveillance, the collective term for capturing early or weak signals of macro-level behaviors and encoding them in quantitative terms, such as the number of ambulance dispatches or the volume of headache remedies sold over the counter in a particular period [21], will likewise constitute a form of sampling. It is difficult to create an efficient and cost-effective system for syndromic surveillance [22,23], but it may contribute towards health goals from a policy point of view in many countries, especially those in which digital data flows and reporting systems are already in place [24]. The aforementioned app may thus be stream-sampled in real time, but the data amassed in its back-end may also undergo data analysis, be subjected to some form of information fusion or synthesis, and finally be made sense of.

Concerns about the individual’s right to privacy have prompted policymakers and opinion leaders to accept the participation of individuals in healthcare decisions about them (‘no decision about me without me’). In many countries, a patient has a smartphone handy at all times during a hospital visit or stay. Mining, predictive analytics, and several other smart methods are thus at arm’s reach. Online communities, including those with patients undergoing similar treatments or having similar symptoms, are equally easy to query for a second opinion or just for comfort. The most striking return from AI in health is not the AI methods contained in or enabled by a smartphone, however. The use of learning machines for second opinions on diagnoses, subscribed to by hospitals, has now arrived. Coupled with data science techniques, including unsupervised learning, such consultations are an interesting complement to human assessments [25].

But it may not stop there. By combining such an app with micro-level and personal data in turn, mHealth can change the perception of an individual and the surrounding environment. In a techno-optimistic future, this will allow the development of a sphere of valuable information surrounding every connected individual: whatever comes into the sphere is analyzed and the results of the analysis are presented to the individual in real time. If the genome of the individual has been sequenced, important alleles such as ApoE4 can be brought into the picture. Questions that some would deem invasive to privacy and others would welcome without hesitation then become broad enough to include:

1. Does this sandwich contain any ingredients that I am allergic to?
2. Is this water safe to swim in?
3. Is the air in here fresh enough for a two-hour meeting?
4. If I have a banana now, will it benefit me more than if I have it in 20 minutes?
5. Has anyone with a high temperature been in this room recently?
6. Will I sleep well now, in this bed?

While some people view such a sphere as leading to a panopticon [26] in the form of a surveillance society [27], the value to others (especially those with special needs, such as those constantly facing serious health threats to themselves or family) is hard to dispute. A barrier to the innovations coming from research and development in mHealth is the defensive strategy of the companies providing the service infrastructure for these apps. Developing a health app today is easy, but getting it into a store to make it available for download can be problematic. It may require scrutiny for whitelisting, ensuring that the app stores all its data on the encrypted part of the memory of the phone, or that no data are stored on the server side, for example. There may be cultural differences or genetic variations at population level, and since most mHealth is global such differences become difficult to control.

## Discussion

Policymakers work with communities of practice relying on health data, and the practitioners’ communities have a lot of experience. Their practices regarding data usage, and the healthcare context in which health data reside, are very different from the actual data discourse, however. The latter consists of static stores of medical images, electronic health records, administrative and insurance records, etc. The former is a dynamic, unstable, difficult to model, and sometimes very stressful environment full of people in different occupational roles [28]. For a policymaker to become an informed decision maker, this difference must be understood and taken seriously, and the transdisciplinary challenge relevant to such understanding is considerable. What is required is no less than ‘sensemaking in turbulent contexts [to provide for] shared meanings in turbulent times’ [29,p.561], and more generally: ‘Closer collaboration between researchers and policymakers, i.e. research needs to be taken outside the academic institutions and into public health programmes that are close to the supply of and demand for health services’ [30].

Efforts to further connect the unconnected must focus primarily on improving affordability and raising awareness, while leveraging the momentum and scale already achieved in the evolution of mobile technology. Building on Internet access, the other three kinds of access can be pushed by policymakers, through openness and transparency, and by describing and taking examples from the mHealth world to show user value, in particular. Sensemaking is essential for this to happen. Only by finding common vocabularies and terms, and by sharing goals and how they are to be prioritized, can policymakers have the opportunity to take adequate action. There are many areas in which methodological development on the ICT side is necessary for making mHealth services globally available within the coming decade, e.g. sensor technology [31,32].

Privacy is a potential showstopper. There are two plausible scenarios for mHealth privacy regulations. In one, the examples (like the one of a parent of a pregnant girl finding out about the pregnancy through directed commercial information addressed to the daughter, thanks to machine learning-boosted ad campaigns) of poor use push regulations to be stricter and so individual data are pushed back into non-use. In the other, norms will change, albeit slowly, to accept the loss of privacy when motivated by health or safety information value. New and stricter regulations for digital services concerning privacy are expected in 2017 for the European Union, making the first scenario more plausible there. The history of technology, however, seems to favor the second scenario: about a dozen years ago, one of the most prominent department stores in the world enforced a ban on cell phones for their customers. The motivation for the ban, which did not last long, was that cameras and microphones in phones could be used in a privacy-invasive manner (and also document proprietary shop displays and goods with the intent of piracy).

## Conclusion

It would be unreasonable to think that a massive and exponentially growing data discourse would, in its own right, provide all the background information necessary for informed decisions. Online as well as offline, policymakers interact with all the stakeholders: clinicians, first responders, nation-state representatives, company representatives, non-governmental organizations, academic institutions, research institutes, and insurance and reinsurance companies. Any model covering the data discourse must also cover its extensions into the communities of practice, and the interplay between the two. Here, understanding what kinds of health information exist is key. Even the smartest analytics possible is not enough to guarantee user value. The interplay between data and practice requires a process of sensemaking: ‘the process of searching for a representation and encoding data in that representation to answer task-specific questions’ [33,p.296]. This means finding out, for a particular stakeholder, how to best inform this person based on data and the conclusions drawn from them, in a particular context. There is again a social dimension: ‘Evaluation assists sensemaking about policies and programs through the conduct of systematic inquiry that describes and explains the policies’ and programs’ operations, effects, justifications, and social implications.’ [34,p.3]. An underlying proviso is that intelligent data analytics can go part of the way to providing a new micro–macro link, moving from population to individual utility and values. Involving the individuals whom the data is about is an important part of the sensemaking, and of precision medicine. Clarifying the transience and dynamicity of different kinds of data, as well as their sensitivity from a privacy perspective, is an equally important challenge.

For the less privileged, all four kinds of access must be provided in order for ICT to really contribute towards meeting the SDGs. Three out of four in the next decade simply is not good enough.

## Paper context

Based on Ericsson mobility data, the outcome of an Ericsson-commissioned foresight, and public reports on the connection between the SDGs and connectivity, this analysis adds health data analytics characteristics and empirical observations on the user value of mHealth and on ICT in general. Implications include an urgent need for sensemaking and for policymakers to work on the micro- as well as the macro-level.

## References

[1] Ransbotham S, Kiron D, Kirk Prentice P. The talent dividend: analytics talent is driving competitive advantage at data-oriented companies. MIT Sloan Management Review; 2015.

[2] McGraw D, Leiter A. A policy and technology framework for using clinical data to improve quality. Houst J Health Law Policy. 2012;122:137–13.

[3] Fact Sheet: President Obama’s Precision Medicine Initiative The White House. Office of the Press Secretary. 2015 Available from: https://www.whitehouse.gov/the-press-office/2015/01/30/fact-sheet-president-obama-s-precision-medicine-initiative

[4] Personalising health and care European Commission H2020 call PHC-31-2014. 2013 11.

[5] United Nations General Assembly Resolution adopted by the General Assembly on 25 September 2015. 70/1. Transforming our world: the 2030 Agenda for Sustainable Development. 2015 Available from: https://sustainabledevelopment.un.org/post2015/transformingourworld

[6] Marmot M, Goldblatt P Importance of monitoring health inequalities. BMJ. 2013;347:f6576. 24192647 10.1136/bmj.f6576

[7] Khoury MJ, Ioannidis JP Big data meets public health: human well-being could benefit from large-scale data if large-scale noise is minimized. Science. 2014;346:1054. 25430753 10.1126/science.aaa2709 PMC4684636

[8] Boman M Who were where when? On the use of social collective intelligence in computational epidemiology Social Collective Intelligence. 2014:203–225.

[9] The Mobile Economy The groupe speciale mobile association (GSMA). 2015 Available from: http://www.gsma.com/mobileeconomy/2016/global/

[10] Sachs JD, Modi V, Figueroa H, et al. ICT & SDGs. The Earth Institute, Columbia University, and Ericsson. 2016 Available from: https://www.ericsson.com/assets/local/about-ericsson/sustainability-and-corporate-responsibility/documents/ict-sdg.pdf

[11] Ericsson Mobility Report 2015 11 Available from: https://www.ericsson.com/res/docs/2015/mobility-report/ericsson-mobility-report-nov-2015.pdf

[12] Walt G, Shiffman J, Schneider H, et al ‘Doing’ health policy analysis: methodological and conceptual reflections and challenges. Health Policy Plan. 2008;23:308–317. 18701552 10.1093/heapol/czn024 PMC2515406

[13] Boman M Innovation radar: 2015 annual trend report. Technical report, EIT Digital. 2016 Available from: http://www.eitdigital.eu/news-events/publications/

[14] Boman M The joy of mesh. Br Med J. 2008;337:a2500. 19052062 10.1136/bmj.a2500

[15] The real digital divide. Economist 2005 Available from: http://www.economist.com/node/3742817

[16] Connected Society: Mobile connectivity index launch report The groupe speciale mobile association (GSMA). 2016 Available from: http://www.gsma.com/mobilefordevelopment/programme/connected-society/mobile-connectivity-index-launch-report

[17] European Strategy and Policy Analysis System (ESPAS) Global trends to 2030: can the EU meet the challenges ahead? 2015 Available from: http://ec.europa.eu/epsc/sites/epsc/files/espas-report-2015.pdf

[18] HealthMap [cited 2016 10 10]. Available from: www.healthmap.org/site/about

[19] Sindi S, Calov E, Fokkens J, et al. The CAIDE Dementia Risk Score App: the development of an evidence-based mobile application to predict the risk of dementia. Alzheimer’s Dementia: Diagnosis, Assess Dis Monit. 2015;1(3):328-333. 10.1016/j.dadm.2015.06.005 PMC4878198 27239514

[20] Brookmeyer R, Johnson E, Ziegler-Graham K, et al Forecasting the global burden of Alzheimer’s disease. Alzheimers Dement. 2007;3:186–191. 19595937 10.1016/j.jalz.2007.04.381

[21] Crubezy M, O’Connor M, Pincus Z, et al Ontology-centered syndromic surveillance for bioterrorism. IEEE Intell Syst. 2005;20:26–35.

[22] Reingold A If syndromic surveillance is the answer, what is the question? Biosecurity Bioterrorism: Biodefense Strategy, Practice, Science. 2004;1:77–81. 10.1089/153871303766275745 15040185

[23] Cakici B The informed gaze: on the implications of ICT-based surveillance. [PhD thesis]. Computer- and Systems Sciences, Stockholm University; 2013.

[24] Boman M, Cakici B, Guttmann C, et al. Syndromic surveillance in the United Arab Emirates In: Innovations in information technology (IIT). Abu Dhabi: IEEE; 2012 p. 31–35.

[25] Raghupathi W, Raghupathi V Big data analytics in healthcare: promise and potential. Health Inf Sci Syst. 2014;2. 10.1186/2047-2501-2-3 PMC4341817 25825667

[26] Foucault M Surveiller et punir. Paris: Gallimard; 1975.

[27] Cakici B, Sanches P Detecting the visible. the discursive construction of health threats in a syndromic surveillance system design. Societies. 2014;4:399–413.

[28] Weick KE Enacted sensemaking in crisis situations. J Manag Stud. 1988;25:305–317.

[29] Maitlis S, Sonenshein S Sensemaking in crisis and change: inspiration and insights from weick (1988). J Manag Stud. 2010;47:551–580.

[30] Mulley A, Evans T, Binagwaho A Meeting the challenges of providing universal health coverage. BMJ. 2013;347:f6485. 24169092 10.1136/bmj.f6485

[31] Baraniuk RG More is Less: signal processing and the data deluge. Science. 2011;331:717–719. 21311012 10.1126/science.1197448

[32] Huston G The internet of stupid things. Asia-Pacific Network Information Centre (APNIC) Blog. 2015 4 30 Available from: https://blog.apnic.net/2015/04/30/the-internet-of-stupid-things/

[33] Russell DM, Stefik MJ, Pirolli P, et al The cost structure of sensemaking, proc interCHI. New York (NY): ACM; 1993 p. 269–276.

[34] Mark MM, Henry GT, Julnes G Evaluation: an integrated framework for understanding, guiding, and improving policies and programs. San Francisco (CA): Jossey-Bass; 2000.

